# A Recipe for Success: Three Key Strategies Used by Aphids and *Pseudomonas syringae* to Colonize the Phyllosphere

**DOI:** 10.1007/s00248-022-01965-2

**Published:** 2022-01-17

**Authors:** Christian Silva-Sanzana, Maria Victoria Gangas, Diego Zavala, Francisca Blanco-Herrera

**Affiliations:** 1 Centro de Biotecnología Vegetal, Facultad de Ciencias de la Vida, Universidad Andres Bello, Santiago, 8370186 Chile; 2 Millennium Science Initiative Program - Millennium Institute for Integrative Biology (iBio), Santiago, Chile; 3 Center of Applied Ecology and Sustainability (CAPES), ANID, Santiago, Chile

**Keywords:** Aphids, *Pseudomonas syringae*, Phytopathology, Plant defense, Effector proteins, Resistance proteins

## Abstract

Aphids and *Pseudomonas syringae* are a permanent challenge for agriculture, causing severe losses to the crop industry worldwide. Despite the obvious phylogenetic distance between them, both have become predominant colonizers of the plant kingdom. In this study, we reviewed three key steps of spread and colonization that aphids and *P. syringae* have mastered to successfully colonize the phyllosphere. These steps involve (i) plant-to-plant movement for locating new nutritional sources, (ii) disruption and modification of the apoplast to facilitate nutrient acquisition, and (iii) suppression of host defenses through effector proteins. In addition, we will provide insights about the direct interaction between aphids and *P. syringae* and how this yet underrated phenomenon could bring new ecological implications for both organisms beyond their pathogenicity.

## Introduction

Pathogenic microorganisms and phytophagous insects constitute a persistent threat to crops around the world. Aphids and *P. syringae* which have become successful plant-interacting agents in terms of their worldwide distribution, number of host species, and the economic losses they caused [1]. For instance, the pandemic outbreak of *P. syringae* pv. *actinidiae* has devastated worldwide kiwi fruit production and negatively affected the major production countries. The bacterial canker in New Zealand’s kiwifruit orchards had caused economic losses of over US$ 666 million [2]. Regarding aphids, species such as *Myzus persicae* feed on more than 400 different plant species [3], and the estimated economic losses caused by virus-transmitting aphids could reach US$482 million per year in Australia [4].

The effectiveness and efficiency exhibited by aphids and *P. syringae* rely on the accomplishment of three key steps of spread and colonization: (1) plant-to-plant movement that allows them to spread to new hosts and find new nutritional sources, (2) locating or creating entrances to internal host tissues and modifying the apoplast to ease nutrient and water acquisition, and (3) modulating the host immunity pathways to promote colonization through the injection of effector proteins into the cytoplasm of host cells. This paper reviews and compares the different mechanism used by aphids and *P. syringae* in order to accomplish these three critical steps of spread and colonization that support their evolutionary success in colonizing the plant kingdom despite their phylogenetic distance.

In addition, we provide insights into the underexplored phenomena concerning the ecological roles of aphids and *P. syringae* beyond their pathogenicity. These phenomena include the possibility of aphid honeydew to promote the spread and proliferation of plant-associated microorganisms in the phyllosphere and the entomopathogenic capacity of *P. syringae* strains which infect and kill different species of aphids.

## Step One: Plant-to-Plant Movement

**Fig. 1:**
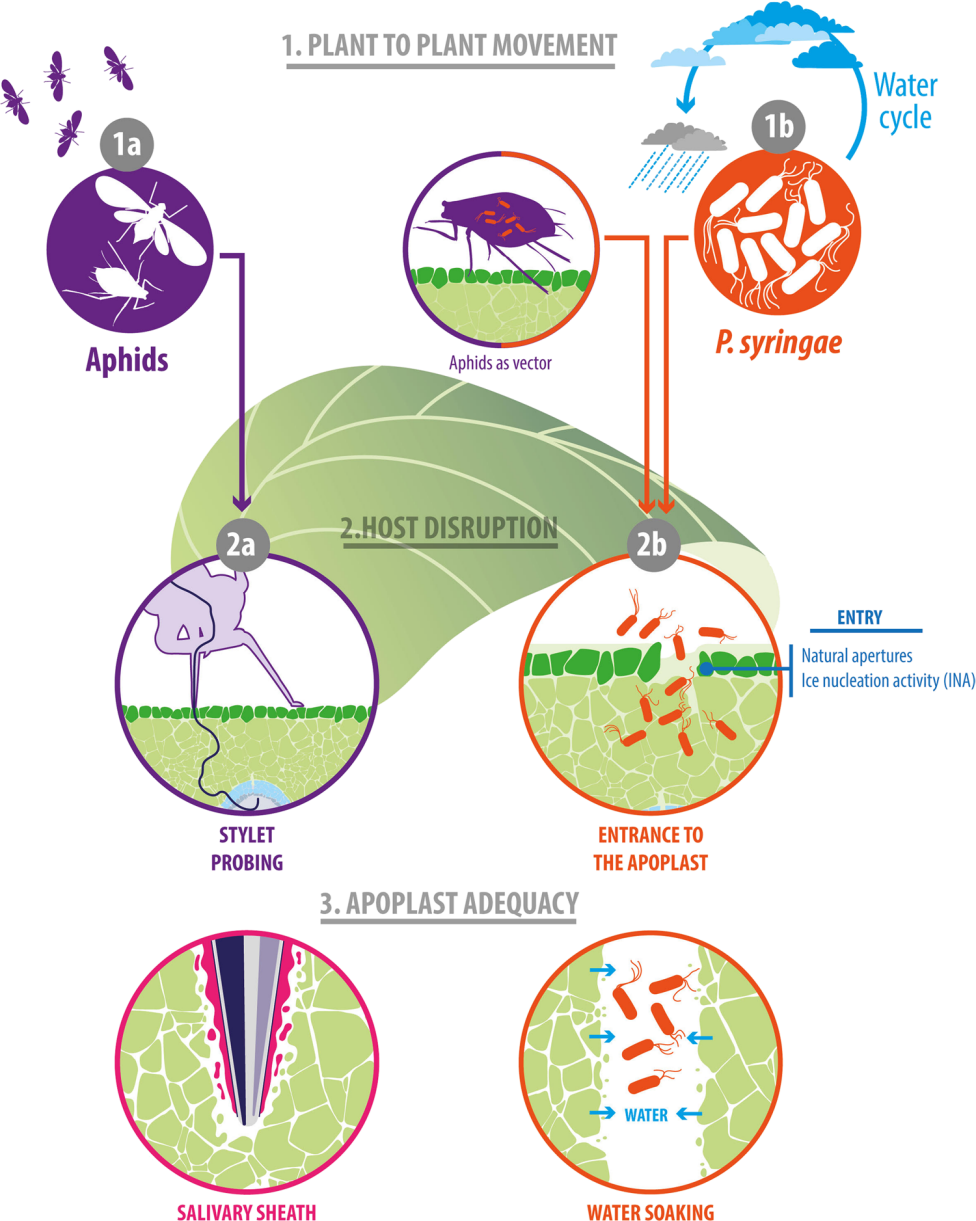
Plant-to-plant movement, host disruption, and apoplast modification

Plant-to-plant movement is critical for herbivore insects and phytopathogens because it facilitates the discovery of new nutritional resources and therefore ensures species survival. In the case of aphids, they are able to move from one plant to another autonomously through their ability to fly. By contrast, *P. syringae* requires a vector or vector agents to achieve such movement. This is provided, for example, by the water cycle and probably by insects (Table 1, Fig. 1). Thus, although the plant-to-plant movements used by aphids and *P. syringae* are clearly different, both have evolved to master their own strategies and become an efficient phyllosphere colonizer, as demonstrated by their worldwide distribution and preponderance.

**Table 1:** General comparison between aphids and *P. syringae*

Spread and colonization step		*Pseudomonas syringae*	Aphids
1. Plant-to-plant movement		Requires a vector to move (Water cycle, insects, etc.)	Winged aphids move autonomously through their ability to fly
2. Host disruption and apoplast modification	Host penetration	Use natural entrances such as stomata and wounds to enter the apoplast. Alternatively, *P. syringae* can create entrances by causing freeze injuries through INA	Stylet probing allows aphids to enter the host and ingest phloem sap, causing minimal mechanical damage to plants
	Apoplast modification	Induce an aqueous environment that promotes bacterial growth	Salivary secretions of aphids modify the apoplast to stabilize the stylet and properly feed
3. Suppression of host defenses	Effector delivery system	The bacterium produces the injectosome called type III secretion system (T3SS) which penetrates the cell wall and translocates effectors directly into the cytoplasm of the host cell	Aphids inject salivary effectors directly into the cytoplasm of host cells through meticulous punctures performed with their stylets
	Function of effector proteins	There are several highly characterized effectors, such as AvrB, AvrPphB, AvrPto, AvrRpm1, HopAI1, HopI1, HopM1, HopZ1. Their main function is to block the signaling cascade downstream the recognition of PAMPs and disrupt the cytoskeleton structure	There is a limited number of characterized effectors, mainly in the aphid Myzus persicae, such as MpC002, Mp1, Mp2, Mp10. Although the specific function remain unknown, their expression is critical for the colonization performance of aphids

### Host Finding and Plant-to-Plant Movement of Aphids

Winged aphids emerged from dense colonies have the capacity to perform exploratory flights and find a new host plant (Fig. 1). To this, the winged explorer evaluates visual, olfactory, and gustatory stimuli to define a proper host. Studies of aphid vision reported that *M. persicae* has visual sensitivity in the spectral regions where plant reflectance is altered when facing stresses [5–7]. This suggests that aphids can visually discriminate stressed from unstressed plants. Interestingly, Hendry et al. (2018) reported that pea aphids (*Acyrthosiphon pisum*) avoided settling on bean leaves on which *P. syringae* strains were growing epiphytically [8]. In addition, the article showed that the molecule pyoverdine synthesized by *P. syringae* emitted fluorescence in the visible range of aphid. This suggest that aphids could be using visual cues to avoid leaves epiphytically colonized by *P. syringae* which is particularly relevant considering that certain *P. syringae* strains can infect and kill different species of aphids [9–11]. This phenomenon adds a new and previously underrated layer of information since a tripartite interaction could be occurring between aphids, *P. syringae*, and plants. Although field experiments are needed to deeply understand this tripartite interaction, it is possible that, in some extent, the bacteria could be influencing the distribution of aphids in phyllosphere.

Besides visual signals, aphids use olfactory cues to locate host plants. Their complex olfactory perception system allows aphids to discriminate between host and nonhost plants, even among closely related species [12]. After the visual and olfactory evaluation, the explorer aphid assesses the internal composition of plants. Using their stylets, aphids carefully probe host tissues, allocate sieve elements, and ingest phloem sap (Fig. 1) [13]. At this stage, phloem composition, presence of toxic compounds, and species-specific defenses are the main factors determining host acceptance. Finally, if the sum of the stimuli displayed by a plant corresponds to a suitable host, its acceptance by the explorer aphid is confirmed by the first offspring deposition, which induces subsequent colonization [3].

### Host Finding via the Water Cycle and Insects: Plant-to-Plant Movement of P. syringae

*P. syringae* is present in the entire water cycle, including in clouds, rainfall, snowfall, seawater, and even soil water flow [14–17]. This is evidence for the spread of *P. syringae* throughout the water cycle (Fig. 1).

The environmental conditions of clouds are highly challenging for organisms because of periodic high temperature differences and high radiation [18]. However, despite these adverse conditions, *P. syringae* is one of the most abundant cultivable species present in clouds [14]. Strains of this bacterium were not affected when subjected to high concentrations of H_2_O_2_ or prolonged sunlight exposure [18], which attests to its high preponderance in clouds and its resilience throughout the entire water cycle. Furthermore, *P. syringae* could be a key component in cloud formation through their role in facilitating ice nucleation activity (INA; [19]). The capacity to form ice-crystal nuclei by *P. syringae* is determined by a single gene (inaZ) that encodes a protein that serves as a catalyzer nucleus to array liquid water, promoting its phase change (crystallization). Thus, this process of water condensation through INA could be what enables *P. syringae* to move through the entire water cycle, facilitating its propagation and colonization of distant host plants.

In addition to the water cycle, other factors might aid in the spread of this microorganism. For example, studies on insect-mediated vectoring have revealed that pollinators (*Apis mellifera* and *Bombus terrestris*) are associated with *P. syringae* pv. *actinidiae* (Psa) when they interact with infected kiwi plants [20]. In addition, it is reported that *P. syringae* can use aphids as an alternative host under laboratory conditions [9–11]. It is reported that *P. syringae* strains epiphytically grown on fava bean plants are ingested by pea aphids and replicate inside the insect leading to its infection and subsequent death. Moreover, Smee et al., 2021 showed that certain *P. syringae* strains can infect and kill five different aphid species of agricultural importance including *M. persicae*. Because of this, Stavrinides et al. (2009) postulate the idea that *P. syringae* could be using aphids as vector since the honeydew excreted by the infected aphids could spread the bacteria over the leaf surface of healthy plants. However, field condition assays are needed in order to test whether this phenomenon occurs in natural ecosystems.

### Highlights Box 1. Plant-to-Plant Movements

- Aphids and *P. syringae* have developed efficient strategies to spread and find new host plants. Although the specific mechanism by which they move from one plant to another are very different, both have mastered this step of spread, being a key milestone for their success as plant colonizers.
- Aphids autonomously move by flying. During the exploratory flights of winged individuals, the aphid evaluates visual, olfactory, and gustatory cues from surrounding plants to find a suitable host and establish a new colony
- *P. syringae* uses the water cycle as the main pathway for spreading throughout the phyllosphere. In addition, increasing evidence suggests that *P. syringae* strains could use aphids and other insects as vectors for plant-to-plant movement.
- The complexity of the tripartite interaction between plants, *Pseudomonas*, and aphids is indicated by the fact that aphids use visual cues to avoid the fluorescence emitted by epiphytic *P. syringae* and that *P. syringae* strains infect aphids and could be using them as a vector. However, experiments in field condition are needed to understand the real ecological implication of this phenomenon.

### Step Two: Host Disruption and Apoplast Modification

**Fig. 2:**
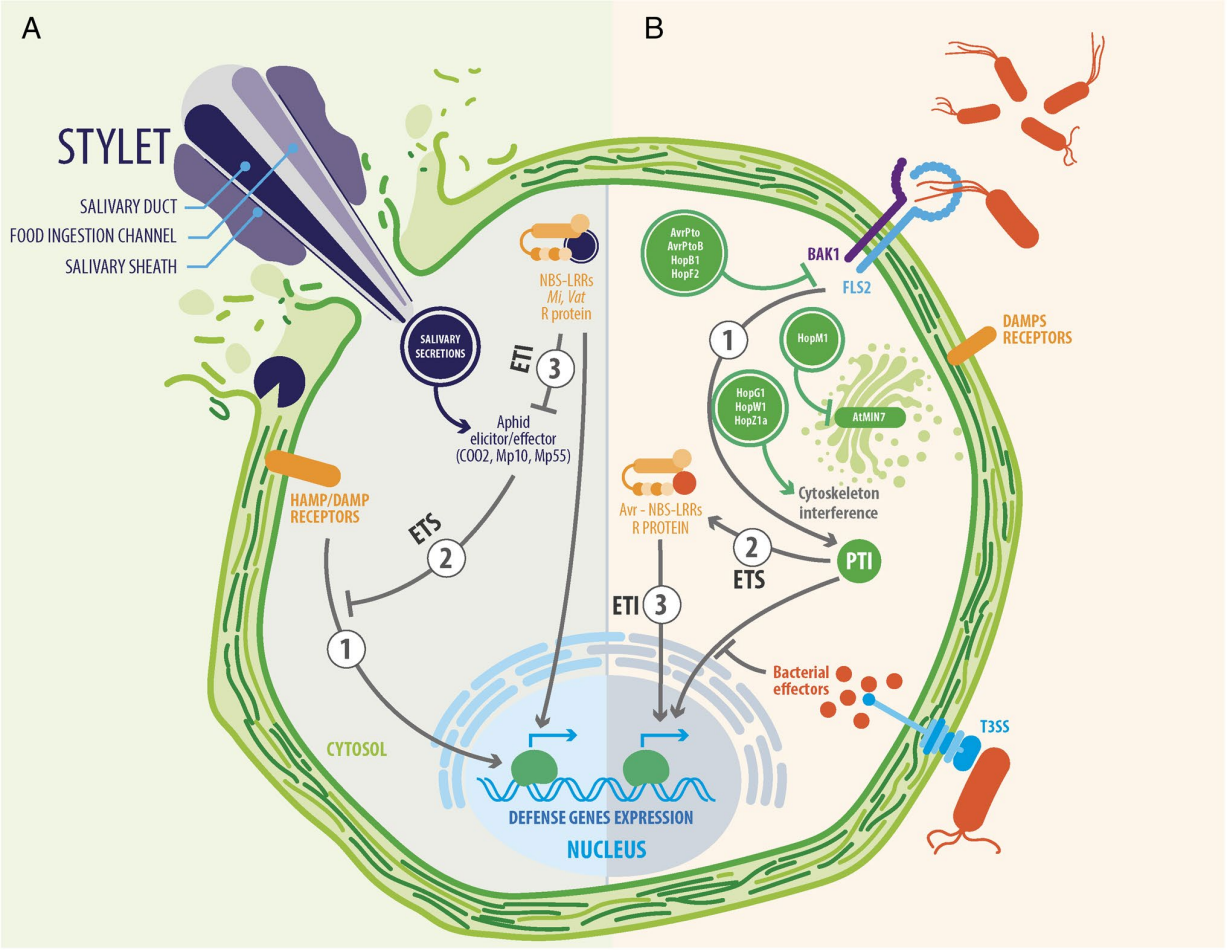
Suppression of host immunity by aphids and *P. syringae*

Aphids and *P. syringae* have evolved their own mechanisms to access internal tissues of host plant. Although these mechanisms are very different, both plant invaders share a common objective which is to enter the host and modify apoplast to ease feeding and replication (Table 1). First, aphids directly disrupt host tissues with their stylet. Next, a salivary sheath is produced around the stylet, creating a stabilizing structure in the apoplast that allow them to feed properly (Fig. 2a). Likewise, *P. syringae* can access the apoplast through natural apertures or creating entrances by causing freeze injuries through its INA. Upon entering the tissue, the bacteria induce water-soaking of the apoplast, which increases water availability and hence facilitates bacterial proliferation (Fig. 2b).

### Creating the Entrance: Host Penetration and Probing of Aphids

Aphids probe and penetrate the host plant with their stylet to obtain phloem sap. The stylet is often classified as a piercing-sucking element. However, this term understates the complexity of this organ. The stylet comprises four substructures that shape two inner and independent channels of salivation and feeding running parallel to two outer neuronal channels containing mechanoreceptor dendrites [13]. This high level of specialization allows aphids to meticulously maneuver the stylets through cell walls and intercellular pathways until a single sieve element is punctured to ingest sap. The specific feeding strategy of aphids leads to a lower damage-associated response than that of other herbivore insects.

Stylet probing is a combined mechanistic and molecular process in which salivary secretions play a leading role. Upon host penetration, a continuous gelling saliva sheath is produced around the stylet, modifying the surrounding apoplast environment to provide stability during intercellular probing and subsequent feeding. The relevance of this tunnel-like structure was illustrated by Will and Vilcinskas (2015), where they silenced the expression of the salivary structural sheath protein and observed a substantial reduction in the time spent by pea aphids on performing probing and sap ingestion [21]. In addition to the saliva sheath, aphids inject watery saliva directly into the cytoplasm of parenchyma cells and sieve elements before sap ingestion to deliver effector proteins that modulate host defense signaling and counteract phloem clogging (Fig. 2; [22]).

### *P. syringae*: Locating or Creating an Entrance to the Apoplast

Before host entrance, *P. syringae *must survive the hostile and nutrient-poor environment of the leaf surface. Here, honeydew deposited by aphids on the leaf surface could be used as a carbon source by *P. syringae* [9] (Fig. 1). Stavrinides et al. (2009) proposed that the sugar-rich honeydew of aphids may enhance bacterial survival on the leaf surface [9]. This hypothesis was subsequently tested by Smee et al. (2019). In their study, *P. syringae* strains exhibited enhanced leaf proliferation on leaves with honeydew [23]. Similarly, Stadler and Müller (1996) reported that phyllosphere microorganisms, including bacteria, yeasts, and fungi, consumed the nutrient-rich honeydew excreted by aphids, leading to an increased population of microorganisms over the aphid-infested leaves [24]. Thus, aphids may play a crucial and underestimated role in the proliferation and spread dynamics of phyllosphere microorganisms, including *P. syringae.*

Once *P. syringae* has detected a natural entrance such as stomata and wounds, the bacteria move toward the entrance and get access to the apoplast. Alternatively, if the environmental conditions are suitable, INA^+^ strains of *P. syringae* can create entrances by generating ice crystals on the leaves, causing frost injuries and thereby enabling bacteria to enter the host [25]. Upon entering the apoplast, *P. syringae* establishes an aqueous environment that promotes bacterial growth [1]. The process of water-soaking of the apoplast is attributed to the function of the bacterial effector HopM1 that targets and degrades the host protein MIN7, which is involved in vesicle trafficking in *Arabidopsis* [26]. Loss-of-function of MIN7 results in the spontaneous occurrence of water-soaking spots in *Arabidopsis* leaves, suggesting that HopM1 could be inducing MIN7 degradation to destabilize the plasma membrane of host cells to create an aqueous apoplast that promotes bacterial growth [1].

### Highlights Box 2. Host Disruption and Apoplast Modification

- Stylet probing allows aphids to enter the host and ingest phloem sap, causing minimal damage to plants.
- Salivary secretions of aphids modify the apoplast to stabilize the stylet and modulate the defense response of the plant (Fig. 2).
- *P. syringae* uses natural entrances such as stomata and wounds to enter the apoplast. Alternatively, *P. syringae* can create entrances by causing freeze injuries through INA.
- The HopM1 effector protein allows *P. syringae* to water-soak the apoplast, increasing water availability and supporting bacterial growth.

## Step Three: Suppression of Host Defenses

Effective colonization of plants requires the pathogen to counteract, tolerate, or interfere with host defenses to prevent being deterred or destroyed. Both aphids and *P. syringae* can modulate the defense response of plants through the direct injection of effector proteins into the cytoplasm of host cells. These effector proteins block the signaling of defensive pathways triggered by plants, thereby promoting colonization (Table 1, Fig. 2).

### Effector-Triggered Susceptibility

The first layer of plant immunity involves the triggering of a defensive response upon perceiving common pathogen-associated molecular patterns (PAMPs) such as flagellar peptides. The binding of PAMPs to the pattern recognition receptors (PRR) commonly localized at the cell membrane initiates a signaling cascade that impedes pathogen proliferation. Thus, a defense response called pattern-triggered immunity (PTI) includes callose deposits, accumulation of reactive oxygen species (ROS), and stomatal closure, among other responses. To counteract such immune responses, both aphids and *P. syringae* have complex effector protein repertories capable of suppressing PTI. *P. syringae* strains harbor a type III secretion system. This syringe-like structure allows the injection of effector proteins into the cytoplasm and suppresses host defense pathways [27]. For instance, the effector proteins AvrPto, AvrPtoB, HopB1, and HopF2 block the signaling cascade downstream the recognition of PAMPs [28]. The AvrPto effector from *P. syringae* pv. tomato DC3000 induced the degradation of the flagellin receptor FLS2 to suppress PTI responses (Fig. 2; [29]). Another element of *P. syringae* pathogenesis is the disruption of cytoskeleton, which is targeted by HopW1, HopG1, and HopZ1a effectors [30] (Fig. 2). Thus, disrupting key processes of plant cells associated with division, growth, vesicle transport, defense responses, and even regulation of stomatal opening [31].

Although knowledge regarding aphid effectors is not as developed as that regarding *P. syringae* effectors, several studies have demonstrated that salivary proteins injected by aphids into the host cells modulate host defense. For example, Elzinga et al. (2014) reported that the *M. persicae* salivary protein Mp55 suppressed PTI responses in *Arabidopsis* (Fig. 2), and the expression of this salivary protein in *Arabidopsis* caused a substantial reduction in callose deposition and ROS accumulation upon aphid infestation [32]. As expected, silencing Mp55 in *M. persicae* reduced aphid performance when aphids feed on *Arabidopsis*, *N. tabacum*, and *N. benthamiana* [32]. In another example, the silencing of the salivary protein C002 of pea aphids caused a substantial reduction in survival and the capacity to maintain sap ingestion [33]. Wang et al. (2021) reported that the salivary protein Mp1 of *M. persicae* interacted with the phloem protein AtPP2-A1 of *A. thaliana* [34]. AtPP2-A1 corresponds to the class of lectin proteins, which play crucial roles in plant defense against insects. The silencing of *Mp1* in *M. persicae* led to a substantial reduction in the phloem ingestion time of aphids as well as reduced fertility and survival [34]. Thus, the salivary effector Mp1 could be suppressing the insecticidal effect of AtPP2-A1, promoting plant colonization.

### Effector-Triggered Immunity

To counteract effector-triggered susceptibility (ETS), plants have evolved a second layer of immunity where the key elements are resistance (R) proteins, whose main feature is to share nucleotide-binding sites and leucine-rich repeat (NBS-LRR) domains. These R proteins recognize pathogen effectors, triggering a more robust defense response called effector-triggered immunity (ETI) that prevents the dissemination of pathogens to systemic tissues. For instance, *P. syringae* strains that carry the AvrPto effector leads to ETS on nonhost plants, but resistant tomato plants expressing the R protein Prf recognize this effector and trigger ETI against the bacteria [35].

Regarding aphids, tomato plants carrying the gene *Mi-1*, encoding for an R protein with a high similarity to Prf, are resistant to the aphid *Macrosiphum euphorbiae* [36]. Similarly, melon plants carrying the *Vat* gene encoding an NBS-LRR protein exhibited enhanced resistance to the melon aphid *A. gossypii* [37].

### Highlights Box 3. Suppression of Host Defenses and Colonization

- Both aphids and *P. syringae* “inject” effector proteins into the cytoplasm of host cells to modulate plant defense and promote the colonization process.
- Plants have developed resistance against aphids and *P. syringae* through R proteins, which recognize pathogenic effectors and trigger a robust defense response.

## Final Remarks and Remaining Challenges

Salivary proteins of aphids are essential for their feeding and manipulation of plant defense. However, their specific targets and mechanisms remain scantly explored. Thus, future research should focus on understanding the mechanism underlying their salivary effectors to improve existing or create new aphid management strategies for crop production.

Although *P. syringae* is a well-studied plant pathogen, its capacity to nucleate ice crystals and its presence throughout the entire water cycle require a complete re-evaluation of the environmental impact and ecological roles of this bacterium.

Honeydew excreted by aphids and other phloem-feeding insects constitutes an abundant source of carbohydrates for microorganisms. This feeding relationship needs to be investigated more comprehensively to facilitate deeper understanding of its impact on the distribution and abundance of bacteria, fungi, and yeast throughout the phyllosphere.

Both aphids and *P. syringae* have been studied mainly in their role as agricultural pests. However, their roles in shaping ecosystems remain underrated. Multidisciplinary studies encompassing molecular biology, pathology, and ecology would aid in providing a more detailed account of the ecological relationships and systemic relevance of these organisms.

The figure illustrates (1) plant-to-plant movement, (2) host disruption, and (3) apoplast modification for aphids and *P. syringae*. More specifically, the following items are depicted: (1a) explorer aphids evaluate the surrounding phyllosphere, locating a host for establishing a new colony. (1b) *P. syringae* uses the water cycle and insects such as aphids to facilitate movement from one plant to another. (2a) Aphids directly create entrances to the internal tissues of the plant by penetrating the tissue with their stylets. (2b) *P. syringae* enters the apoplast through natural apertures such as stomata and wounds. Alternatively, INA + strains of *P. syringae* cause freeze injuries through which the bacteria can enter the tissue. (3a) Upon penetrating the intercellular spaces, the stylet produces a salivary sheath, modifying the surrounding apoplast to provide stability during intercellular probing and sap ingestions. (3b) Upon entering the host, *P. syringae* disrupts the host cell membranes, causing the water-soaking of the apoplast. This increases water and nutrient availability and promotes bacterial growth.

The figure illustrates (1) the recognition of common features of biotic threats (PAMPs; e.g., flagellar proteins and chitin) through PRRs (e.g., FLS2). The recognition of these patterns initiates a set of broad defensive responses (PTI). (2) However, plant pathogens have evolved mechanisms to suppress PTI through effector proteins (e.g., AvrPto and Mp55), which block PRR signaling, causing ETS. (3) In turn, plants have developed resistance proteins (e.g., Prf and Vat) that recognize pathogen effectors and elicit a robust defense response against the attacking agent, leading to ETI.

## Data Availability

Data sharing is not applicable to this article as no new data were created or analyzed in this study.
